# Utility of Intracardiac Echocardiography to Diagnose Infective Endocarditis

**DOI:** 10.1177/2324709618822075

**Published:** 2019-01-15

**Authors:** Ying Chi Yang, Thein Tun Aung, Sarah Khan, Abdul Wase

**Affiliations:** 1 University of Iowa Hospitals and Clinics, Iowa City, IA, USA; 2 North East Ohio Medical University, Rootstown, OH, USA; 3 Wright State University, Dayton, OH, USA

**Keywords:** intracardiac echography, prosthetic valve, infective endocarditis

## Abstract

Infective endocarditis (IE) can lead to significant morbidity and mortality without appropriate treatment. Modified Duke Criteria are accepted by many professional societies to establish the diagnosis of IE, and cardiac imaging is one of the major diagnostic criteria. Transesophageal echocardiography is an algorithmic escalation to diagnose IE when transthoracic echo does not appreciate a positive finding. In patients with contraindications to transesophageal echocardiography, cardiac magnetic resonance imaging, cardiac computed tomography (CT), cardiac CT angiography, and fluorodeoxyglucose positron emission tomography with CT or CT angiography may be alternative diagnostic tools. However, these imaging modalities have their own limitations such as local unavailability, the presence of non–magnetic resonance imaging compatible implants, or impaired renal function. Intracardiac echocardiography could be a considerable alternative under those circumstances.

## Introduction

Undiagnosed or delayed diagnosis of infective endocarditis (IE) can lead to significant morbidity and mortality. Mortality rate from untreated IE may be as high as 50%. The Modified Duke Criteria are accepted by many professional societies to establish the diagnosis of IE, and the role of cardiac imaging is one of the major diagnostic criteria. We present a case of IE in which routine imaging modalities were limited due to the patient’s comorbid condition, and we utilized an unconventional imaging modality to establish the diagnosis.

## Case Presentation

A 72-year-old male with a history of bioprosthetic aortic valve replacement was admitted for generalized weakness and fatigue. He was found to have anemia with positive fecal occult blood and subsequently received upper esophagogastroduodenoscopy revealing an obstructive esophageal cancer. Given the presence of the prosthetic aortic valve and an episode of bradycardia that occurred during colonoscopy, the cardiology team was involved in the patient’s care.

During his hospital stay, the patient had intermittent fever and leukocytosis. Blood cultures were positive for *Staphylococcal* species. Appropriate antibiotics failed to improve his fever. A transthoracic echocardiogram (TTE) revealed no abnormalities of bioprosthetic aortic valve except mild aortic regurgitation.

**Figure 1.**
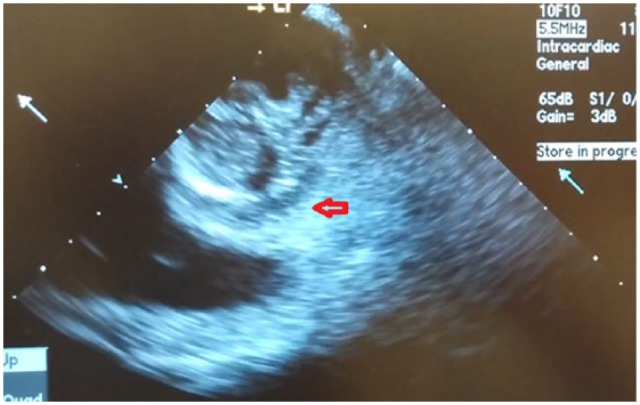
Short axis view of the aortic valve reveals the perivalvular abscess (red arrow) around the prosthetic aortic valve.

**Figure 2.**
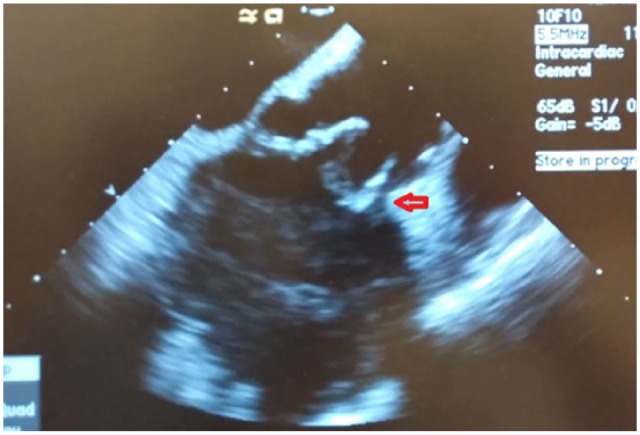
Longitudinal view of the aortic valve reveals the perivalvular abscess (red arrow). The rocking movement of the aortic prosthesis was noted in the live images.

The TEE was not feasible due to the advanced obstructive nature of esophageal cancer. Intracardiac echo (ICE) was attempted in this patient to establish a prompt diagnosis and institute an appropriate treatment. The AcuNav, 8 Fr intracardiac echography probe (Siemens AG, Munich, Germany) was introduced into the right femoral vein and then advanced into the right atrium and subsequently into the right ventricle. The bioprosthetic aortic valve was visualized in both short axis and longitudinal views (Figures 1 and 2).

The images disclosed the perivalvular aortic root abscess as well as a rocking motion of the bioprosthesis with moderate aortic regurgitation. All these findings were consistent with complicated IE. Periaortic root abscess was drained by an urgent surgical intervention and the infected bioprosthetic valve was also replaced. The patient was referred for urgent surgery, where the perivalvular aortic root abscess was drained, and subsequently the infected bioprosthetic valve was replaced.

## Discussion

IE is diagnosed by Modified Duke Criteria, and the role of cardiac imaging is one of the major diagnostic criteria. TTE and TEE are the mainstay imaging modalities.

The sensitivity and specificity of TTE in detecting valvular vegetations or perivalvular abscesses associated with native valve endocarditis are 28% to 63% and 91% to 99%, respectively, and for TEE are 87% to 94% and 91% to 100%, respectively.^1-4^ TTE diagnoses more sensitively for the valvular vegetations, which are greater than 6 mm but the sensitivity decreases to 25% when the vegetation size is less than 5 mm.^4^ TEE can offer a closer view to the heart via the esophageal axis and it could detect a vegetation size of 1 mm or larger. However, the role of TEE is limited in patients with certain comorbid conditions such as esophageal cancer, extensive esophageal ulceration, Zenker’s diverticulum, or anatomic abnormalities of the esophagus, since the probe would not be able to pass down or the risk of iatrogenic esophageal perforation is high.

Cardiac magnetic resonance imaging (MRI), cardiac computed tomography (CT), cardiac CT angiography (CTA), and fluorodeoxyglucose positron emission tomography (PET) with CT or CTA (FDG PET/CT or FDG PET/CTA) are alternative diagnostic tools in those patients who have a contraindication to TEE.^5-7^ However, the roles of those imaging modalities may sometimes be limited as they often depend on other factors such as the local availability of the imaging machines or experienced interpreters, the presence of non-MRI compatible implants, or impaired renal function.^8^

The utility of ICE has been established for real-time visualization of intracardiac structures during complex ablation procedures to minimize the risks of unwanted complications (ie, pericardial tamponade). ICE is also used to diagnose cardiac device-related infective endocarditis.^9,10^ However, the role of ICE in diagnosing native or prosthetic valves related IE has not been well established to date as only one related study and a case report have been published.^11,12^ We utilized ICE in our patient to establish a prompt diagnosis and institute an appropriate treatment.

In our case, ICE not only disclosed the perivalvular aortic abscess with vegetations on the bioprosthetic valve but also revealed the rocking motion of the bioprosthesis confirming the diagnosis of the prosthetic aortic valve endocarditis.

## Conclusion

In patients with contraindications to TEE, noninvasive imaging modalities such as cardiac MRI, CT/CTA, or FDG CT/CTA may be alternative diagnostic tools for IE. Unfortunately, these imaging modalities have limitations such as local unavailability, the presence of non-MRI compatible implants, impaired renal function, and so on. Intracardiac echocardiography could be a considerable alternative under those circumstances.

## References

[1] Daniel WG Mugge A Martin RP et al Improvement in the diagnosis of abscesses associated with endocarditis by transesophageal echocardiography. N Engl J Med. 1991;324:795-800. 1997851 10.1056/NEJM199103213241203

[2] Shapiro SM Young E De Guzman S et al Transesophageal echocardiography in diagnosis of infective endocarditis. Chest. 1994;105:377-382. 8306732 10.1378/chest.105.2.377

[3] Shively BK Gurule FT Roldan CA Leggett JH Schiller NB. Diagnostic value of transesophageal compared with transthoracic echocardiography in infective endocarditis. J Am Coll Cardiol. 1991;18:391-397. 1856406 10.1016/0735-1097(91)90591-v

[4] Erbel R Rohmann S Drexler M et al Improved diagnostic value of echocardiography in patients with infective endocarditis by transoesophageal approach. A prospective study. Eur Heart J. 1988;9:43-53. 3345769

[5] Feuchtner GM Stolzmann P Dichtl W et al Multislice computed tomography in infective endocarditis. Comparison with transesophageal echocardiography and intraoperative findings. J Am Coll Cardiol. 2009;53:436-444. 19179202 10.1016/j.jacc.2008.01.077

[6] Dursun M Yılmaz S Yılmaz E et al The utility of cardiac MRI in diagnosis of infective endocarditis: preliminary results. Diagn Interv Radiol. 2015;21:28-33. 25430531 10.5152/dir.2014.14239 PMC4463365

[7] Fagman E Perrotta S Bech-Hanssen O et al ECG-gated computed tomography: a new role for patients with suspected aortic prosthetic valve endocarditis. Eur Radiol. 2012;22:2407-2414. 22622348 10.1007/s00330-012-2491-5

[8] Salaun E Habib G. Beyond standard echocardiography in infective endocarditis: computed tomography, 3-dimensional imaging, and multi-imaging. Circ Cardiovasc Imaging. 2018;11:e007626. 10.1161/CIRCIMAGING.118.007626 29555840

[9] Narducci ML Pelargonio G Russo E et al Usefulness of intracardiac echocardiography for the diagnosis of cardiovascular implantable electronic device-related endocarditis. J Am Coll Cardiol. 2013;61:1398-1405. 23500279 10.1016/j.jacc.2012.12.041

[10] Ali S George LK Das P Koshy SKG Intracardiac echocardiography: clinical utility and application. Echocardiography. 2011;28:582-590. 21564275 10.1111/j.1540-8175.2011.01395.x

[11] Bouajila S Chalard A Dauphin C. Usefulness of intracardiac echocardiography for the diagnosis of infective endocarditis following percutaneous pulmonary valve replacement. Cardiol Young. 2017;27:1406-1409. 28322179 10.1017/S1047951117000403

[12] Kolodner DQ Shimbo D Magnano AR. Intracardiac echocardiography in the diagnosis of prosthetic valve endocarditis. Heart. 2007;93:1120. 10.1136/hrt.2006.104091 PMC1955030 17699175

